# An antioxidant specifically targeting mitochondria delays progression of Alzheimer's disease-like pathology

**DOI:** 10.18632/aging.101054

**Published:** 2016-10-06

**Authors:** Natalia A. Stefanova, Natalia A. Muraleva, Kseniya Yi. Maksimova, Ekaterina A. Rudnitskaya, Elena Kiseleva, Darya V. Telegina, Nataliya Kolosova

**Affiliations:** ^1^ Institute of Cytology and Genetics SB RAS, 630090, Novosibirsk, Russia; ^2^ Siberian State Medical University, 634055, Tomsk, Russia; ^3^ Novosibirsk State University, 630090, Novosibirsk, Russia

**Keywords:** Alzheimer's disease, mitochondria, amyloid, synapse, neurodegeneration

## Abstract

Mitochondrial aberrations are observed in human Alzheimer's disease (AD) and in medical conditions that increase the risk of this disorder, suggesting that mitochondrial dysfunction may contribute to pathophysiology of AD. Here, using OXYS rats that simulate key characteristics of sporadic AD, we set out to determine the role of mitochondria in the pathophysiology of this disorder. OXYS rats were treated with a mitochondria-targeted antioxidant SkQ1 from age 12 to 18 months, that is, during active progression of AD-like pathology in these animals. Dietary supplementation with SkQ1 caused this compound to accumulate in various brain regions, and it was localized mostly to neuronal mitochondria. Via improvement of structural and functional state of mitochondria, treatment with SkQ1 alleviated the structural neurodegenerative alterations, prevented the neuronal loss and synaptic damage, increased the levels of synaptic proteins, enhanced neurotrophic supply, and decreased amyloid-β1-42 protein levels and tau hyperphosphorylation in the hippocampus of OXYS rats, resulting in improvement of the learning ability and memory. Collectively, these data support that mitochondrial dysfunction may play a key role in the pathophysiology of AD and that therapies with target mitochondria are potent to normalize a wide range of cellular signaling processes and therefore slow the progression of AD.

## INTRODUCTION

Alzheimer's disease (AD) is a progressive, age-dependent neurodegenerative disorder featuring progressive impairments in memory and cognition and ultimately leads to death [1]. According to the most widely accepted theory, the “amyloid cascade” hypothesis [2], AD arises when amyloid precursor protein (APP) is processed into amyloid-β, which accumulates in plaques. The form of this protein that is most toxic to cells (amyloid-β_42_) promotes tau hyperphosphorylation, which leads to formation of neurofibrillary tangles: the effect directly causing mitochondrial dysfunction, synaptic damage, and neuronal cell death [3–4]. In many ways, the above hypothesis has been highly successful, in that it unites findings from many different investigative approaches to the disease and, most compellingly, helps to explain how mutations in genes *APP*, presenilin 1 (*PSEN1*), or *PSEN2* can cause the familial early-onset form of AD, which accounts for ~5% of all cases [4–5]. Nonetheless, the therapeutic strategies designed to eliminate amyloid-β production as the key precipitating event in Alzheimer's pathogenesis have not been successful [1]. Consequently, the factors that initiate (or affect the risk and onset of) amyloid-β accumulation in sporadic late-onset AD, which accounts for ~95% of all disease cases, remain poorly understood [6].

There is growing evidence that mitochondrial damage and oxidative stress lead to activation of the amyloid-β cascade and, accordingly, the mitochondrial dysfunction is a significant contributing factor of the onset and progression of AD. According to the “mitochondrial cascade hypothesis” [7] amyloid-β is a marker of brain aging, and not a singular cause of AD [8]. Many studies have confirmed that mitochondrial dysfunction is likely to be the leading cause of synaptic loss and neuronal death by apoptosis, representing the most likely mechanism underlying cortical shrinkage, especially in brain regions involved in learning and memory, such as the hippocampus [9]. The mitochondrial changes increase amyloid-β production and cause its accumulation, which in turn can directly exert toxic action on mitochondria, thus aggravating the neurodegenerative processes. Triggering of the “vicious circle” of neurodegeneration leads to tau hyperphosphorylation, synaptic damage, and neuronal cell death and serves as a fatal hallmark event of Alzheimer's pathophysiology. Naturally, the mitochondrial dysfunction is called the missing link separating brain aging and AD [10], and recent studies have provided substantial evidence that therapeutic strategies targeting mitochondria may shed some light on new strategies for treatment of AD [1, 11]. A promising approach to this problem may be the use of antioxidants specifically targeting mitochondria, for example, mitochondria-targeted antioxidants of the SkQ family [11]. These membrane-penetrating cations specifically accumulate in the inner mitochondrial membrane because the mitochondrial interior is the only negatively charged compartment in the cell; such compounds have been shown to have a beneficial effect in a number of age-related diseases [12–15]. In our studies involving senescence-accelerated OXYS rats, we demonstrated that dietary supplementation with plastoquinonyl-decyltriphenylphosphonium (SkQ1) in OXYS rats starting at a young age is clearly pleiotropic in nature, inhibits a large set of typical traits of aging, and suppress age-related diseases [16–23], including development of signs of AD [24–25]. Recently, we showed that OXYS rats simulate key characteristics of the sporadic form of AD [25–27]. More recently, we reported that an age-dependent increase in the levels of amyloid-β_1–42_ and extracellular amyloid-β deposits in the brain of OXYS rats occur later than do mitochondrial structural abnormalities, hyperphosphorylation of the tau protein, synaptic loss, and neuronal cell death [28]. On the basis of our results, we theorized that multiple age-associated degenerative processes may precede the toxic accumulation of amyloid-β, which in turn triggers the final, currently irreversible stage of AD.

In the present study, we explored the role of mitochondria in the brain affected by AD using mitochondria-targeted antioxidant SkQ1 as a possible therapeutic agent for alleviation of the deleterious consequences of this disease in a rat model. To obtain evidence of the hypothesized role of mitochondrial damage in AD, treatment with SkQ1 was started at the progressive stage of AD-like pathology in OXYS rats. Via improvement of the structural and functional state of mitochondria in the hippocampus of OXYS rats by SkQ1, we showed alleviation of structural neurodegenerative alterations, prevention of the neuronal loss and synaptic damage, improvement of the neurotrophic supply, a decrease in the toxic amyloid-β protein levels and in tau hyperphosphorylation, and a recovery of reference memory. All these changes suggest that mitochondrial damage may play a key role in AD.

## RESULTS

### SkQ1 is predominantly localized to neuronal mitochondria in the brain

**Figure 1:**
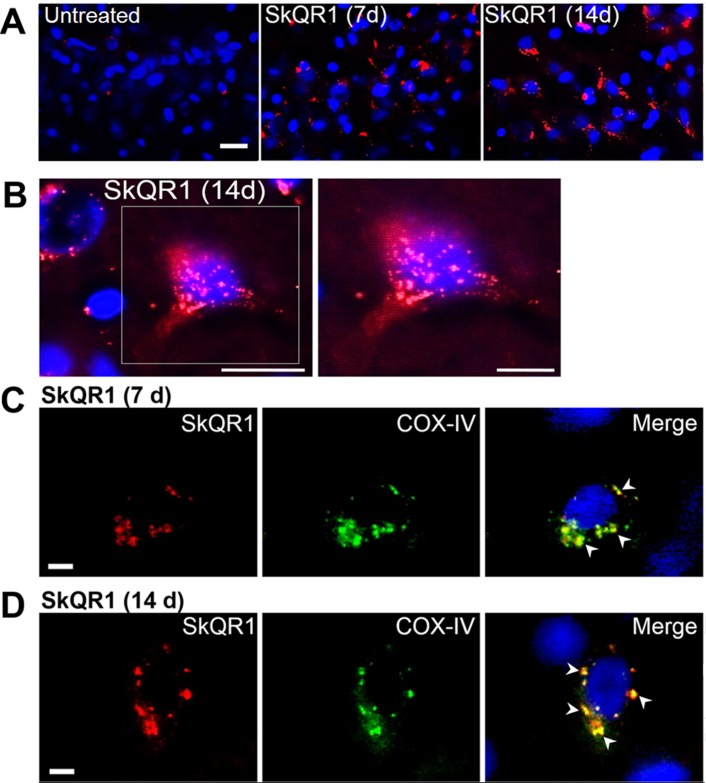
SkQ1 is predominantly localized to (and accumulates in) neuronal mitochondria Four-month-old OXYS rats were treated with SkQR1 (250 nmol/kg) for 7 or 14 days (n = 4) to assess localization and accumulation of SkQ1. (**A**) High intensity of fluorescence signals of fluorescence signals of rhodamine-labeled of SkQ1 (red fluorescence) in neurons of the hippocampus in SkQR1-treated OXYS rats and the absence of specific signals in untreated (control) OXYS rats. The SkQR1 signal increased depending on the duration of treatment with the antioxidant (7 days < 14 day). (**B**) An example of accumulation of SkQR1 (in red) in hippocampal neurons of an OXYS rat. (**C** and **D**) Immunolabeling with an anti-COX-IV antibody (mitochondrial loading control; in green) shows that SkQR1 (in red) is localized to neuronal mitochondria. The arrows show colocalization of SkQR1 and COX-IV. Cell nuclei are stained with DAPI (in blue). The scale bars are 20 μm (**A** and **B**) and 5 μm (**C** and **D**).

We first assessed the localization of SkQ1 in the brain, using a rhodamine derivative of SkQ1, 10(6′-plastoquinonyl) decylrhodamine 19 (SkQR1), which, as shown elsewhere [11, 14], also has neuroprotective properties. We found high-intensity fluorescent signals of SkQR1 in the brain neurons of OXYS rats treated with this antioxidant for 7 or 14 days (n=4). Depending on duration of administration, the signal of SkQR1 increased (7 day < 14 days) and was most noticeable in neurons of the hippocampus (Fig. 1A–D), cortex (Fig. S1A), thalamus, myelencephalon, and cerebellum (Fig. S1B) of OXYS rats, suggesting that SkQ1 accumulates in cerebral neurons. Moreover, using the antibody to COX-IV (cytochrome *c* oxidase subunit 4, mitochondrial loading control), we found that SkQR1 is localized to neuronal mitochondria (Fig. 1C and D), suggesting that this antioxidant may play an important neuroprotective role by regulating and restoring the mitochondrial function.

### SkQ1 retards mitochondrial alterations

**Figure 2:**
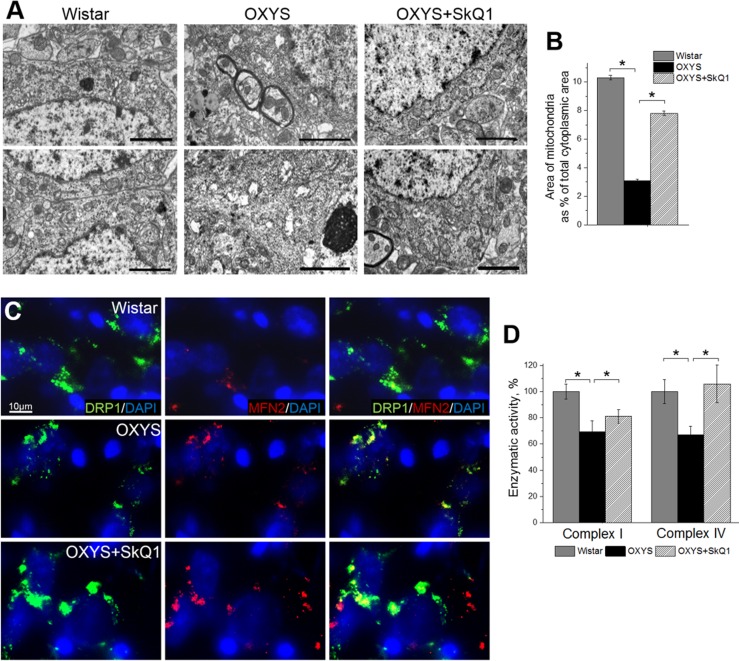
SkQ1 retards mitochondrial alterations in the hippocampus (**A**) Alterations in the ultrastructure of the mitochondrial apparatus of CA1 pyramidal neurons in OXYS rats were attenuated by SkQ1 administration. (**B**) The area occupied by mitochondria within the hippocampal pyramidal neurons as a percentage of the total neuronal area in 18-month-old Wistar rats, untreated OXYS rats, and SkQ1-treated OXYS rats (n=4). Compared to Wistar rats, the untreated OXYS rats had a considerably smaller mitochondrial area. SkQ1 administration increased the mitochondria-occupied portion of the neuronal area. (**C**) Immunolabeling for mitochondrial fission mediator Drp1 (green) and mitochondrial fusion mediator Mfn2 (red) and cell nuclei (DAPI, blue) in the CA3 region of the hippocampus. (**D**) Enzymatic activities of complexes I and IV in the hippocampus of OXYS rats were ~30% lower than those in Wistar rats (p<0.03 and p<0.02, respectively). Enzymatic activity did not change significantly in complex I but increased by ~30% in complex IV (p<0.001) in the hippocampus of SkQ1-treated OXYS rats (n=6). The data are shown as mean ± SEM. Statistical significance (p<0.05) is denoted by the asterisk. The scale bar is 2 μm (**A**).

We next tested whether SkQ1 treatment from age 12 to 18 months (that is, during active progression of AD-like pathology in OXYS rats) affects mitochondrial alterations in the hippocampus. To this end, in OXYS rats, we evaluated the influence of SkQ1 administration on the ultrastructural state of the mitochondrial apparatus in pyramidal neurons of the hippocampal CA1 region, as the most vulnerable region of the hippocampus during the development of AD [29–30]. According to electron-microscopic analysis, in contrast to Wistar rats and SkQ1-treated OXYS rats, untreated OXYS rats showed significant changes in structural organization of the mitochondrial apparatus, such as large intramitochondrial cristae-free areas of low electron density with an uneven contour of the external and internal membranes as well as localization of cristae near the periphery only; the latter picture corresponds to a de-energized state (Fig. 2A). In some locations, we could see contacts of the mitochondria with the nuclear envelope, the endoplasmic reticulum, and Golgi complex. SkQ1 treatment substantially improved the ultrastructure of the mitochondrial apparatus (Fig. 2A) and increased the specific area of mitochondria (Fig. 2B) in neurons in the hippocampal CA1 region of OXYS rats. In addition, in untreated OXYS rats, we seldom found mitochondria touching each other via membranes: this touching can be a sign of fusion or fission and was observed more frequently in Wistar rats and SkQ1-treated OXYS rats. To determine the state of mitochondrial dynamics, we immunostained rat brain tissue for Drp1 (dynamin-1-like protein; mitochondria in fission) and for Mfn2 (mitofusin 2; mitochondria in fusion).

In the hippocampus, we found increased expression of Drp1 and Mfn2 in SkQ1-treated OXYS rats and Wistar rats compared to untreated OXYS rats (Fig. 2C); this result was suggestive of improvement of the mitochondria biogenesis processes in OXYS rats by SkQ1. Finally, using Microplate Assay Kits we found that enzymatic activity of complexes I and IV in the hippocampus of untreated OXYS rats was ~30% lower than that in Wistar rats. Less pronounced interstrain differences in these parameters compared to the scale of detected by electron microscopy mitochondrial destructions, we can assume methodological reasons. The enzymatic activity of the respiratory chain complexes was determined in the isolated mitochondria, in which severely damaged mitochondria do not fall. Administration of SkQ1 slightly but not significantly increased enzymatic activity of complex I and significantly increased enzymatic activity of complex IV in OXYS rats to the level of Wistar rats (Fig. 2D). Taken together, these findings indicate that the mitochondrial alterations involved in progression of AD-like pathology in OXYS rats can be significantly attenuated by SkQ1-driven improvement of mitochondrial function.

### SkQ1 prevents neuronal loss and retards structural neurodegenerative alterations

**Figure 3:**
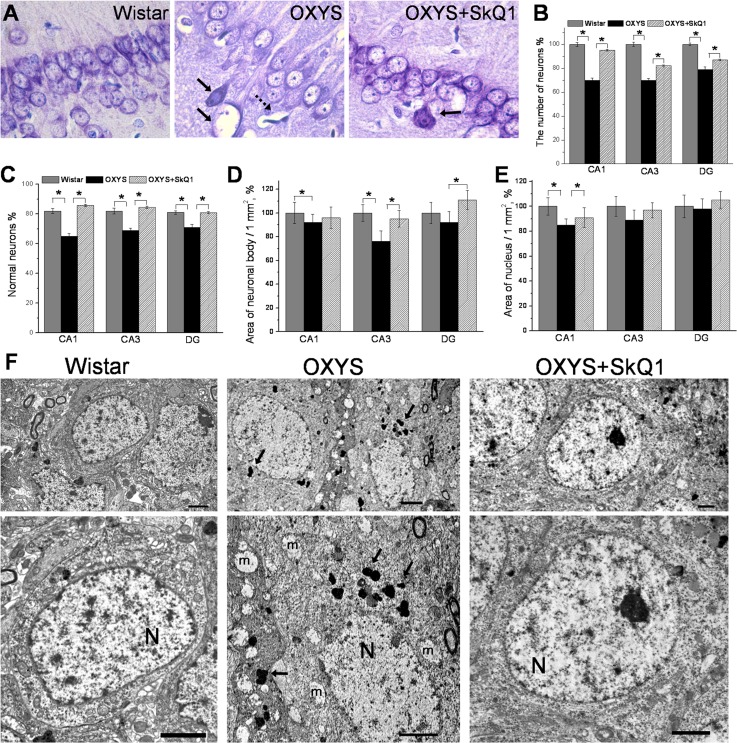
SkQ1 prevents neuronal loss and retards structural neurodegenerative alterations (**A**) Representative 100 × histological Cresyl violet images of neurons of the hippocampal CA1 region in 18-month-old Wistar rats, untreated OXYS rats, and SkQ1-treated OXYS rats (n=4). The damaged neurons are indicated by solid arrows, and a dead neuron is indicated by a dashed arrow. (**B**) Quantification of neuronal populations revealed a significant decrease in neuronal number in the CA1 and CA3 regions and in the dentate gyrus of untreated OXYS rats compared to Wistar rats (p<0.05). Treatment with SkQ1 significantly increased neuronal populations in the hippocampus of OXYS rats (p<0.05). (**C**) The percentages of normal neurons were smaller in the examined hippocampal regions of OXYS rats compared to Wistar rats. Oral SkQ1 administration improved neuronal health in all the hippocampal regions examined in OXYS rats. (**D**) The small area of neuronal bodies in the CA3 region and dentate gyrus of the hippocampus in OXYS rats (n=5) is enlarged in response to SkQ1 treatment (p<0.05). (**E**) The small area of neuronal nuclei in the hippocampal CA1 region of OXYS rats (n=5) is enlarged in response to SkQ1 treatment (p<0.05). (**F**) SkQ1 improved the ultrastructure of pyramidal neurons in the hippocampal CA1 region in 18-month-old OXYS rats (n=4). The electron micrographs show examples of the normal ultrastructure of pyramidal neurons of Wistar rats and SkQ1-treated OXYS rats, and the substantial changes in the structural organization of cellular organelles in the CA1 region of untreated OXYS rats. The figure shows a pyramidal neuron containing a large cluster of lipofuscin granules (indicated by arrows), and massively swollen mitochondria (m). N: nucleus. The scale bars are 2 μm. DG: dentate gyrus. The data are shown as mean ± SEM. Statistical significance (p<0.05) is denoted by the asterisk.

An important and distinct feature of OXYS rats is that these rats show an overt neuronal loss in the hippocampus at the progressive stage of AD-like pathology [27]. When we evaluated the effects of SkQ1 in this crucial period, we stained brain tissue with Cresyl violet to visualize cell nuclei and stereologically quantified the number of neurons in the hippocampus (Fig. 3A). Quantification of neuronal populations revealed a significant decrease in neuronal numbers in the CA1 and CA3 regions and in the dentate gyrus of OXYS rats, whereas SkQ1 treatment prevented this decrease (Fig. 3B). In addition, quantification using stereological counts revealed that the specific proportion of unaffected neurons in the CA1 and CA3 areas in the dentate gyrus of the OXYS hippocampus was lower, whereas the proportion of dead or damaged neurons was higher than that in Wistar rats, in all hippocampal regions (Fig. 3C, Fig. S2). Administration of SkQ1 slowed the progression of degenerative alterations of hippocampal neurons in OXYS rats (Fig. S2): the number of damaged neurons in all the analyzed hippocampal regions in SkQ1-treated OXYS rats was substantially lower than that in untreated OXYS rats, and did not differ from these parameters in Wistar rats. Accordingly, the number of normal neurons in all hippocampal regions of SkQ1-treated OXYS rats was at the level of Wistar rats and higher than in untreated OXYS rats (Fig. 3C). Next, we analyzed the effects of SkQ1 treatment on the size of pyramidal neurons in the hippocampal CA1 and CA3 regions and dentate gyrus of OXYS rats. We found that in OXYS rats in the CA1 region, the average area of neuronal bodies and nuclei—and in the CA3 region, the average area of neuronal bodies—were less than those in the Wistar strain (Fig. 3D, E). SkQ1 treatment increased the area of neuronal bodies in the CA3 region and dentate gyrus of the OXYS hippocampus (Fig. 3D).

Besides, in SkQ1-treated OXYS rats, the area of the nuclei in the CA1 region was larger than that in untreated OXYS rats and was not different from this parameter in Wistar rats (Fig. 3E). Further analyses of the ultrastructural state of pyramidal neurons in the hippocampal CA1 region of the rats revealed significant changes (similar to those showed recently [31]) in the structural organization of the nucleus, nucleolus, Golgi complex, endoplasmic reticulum (Fig. 3F) and mitochondria (Fig. 2B) in OXYS rats compared to the Wistar strain. In addition, in many neurons of OXYS rats, there were numerous vacuoles and phagolysosomes of various sizes and shapes (Fig. 3F). Administration of SkQ1 substantially improved the ultrastructural state of neurons in OXYS rats (Fig. 3F) and according to morphometric analysis, significantly increased specific area of mitochondria (Fig. 2B) and the endoplasmic reticulum, decreased specific area of lysosomes and vacuoles, and slightly but not significantly decreased the specific area of the Golgi complex (Table S1). These data corroborate previous reports of neuronal loss and neurodegenerative changes in the hippocampus of OXYS rats at this age [31] and indicate that SkQ1 treatment prevented the neuronal loss and progression of neurodegeneration in these rats.

### SkQ1 improves the neurotrophic supply

**Figure 4:**
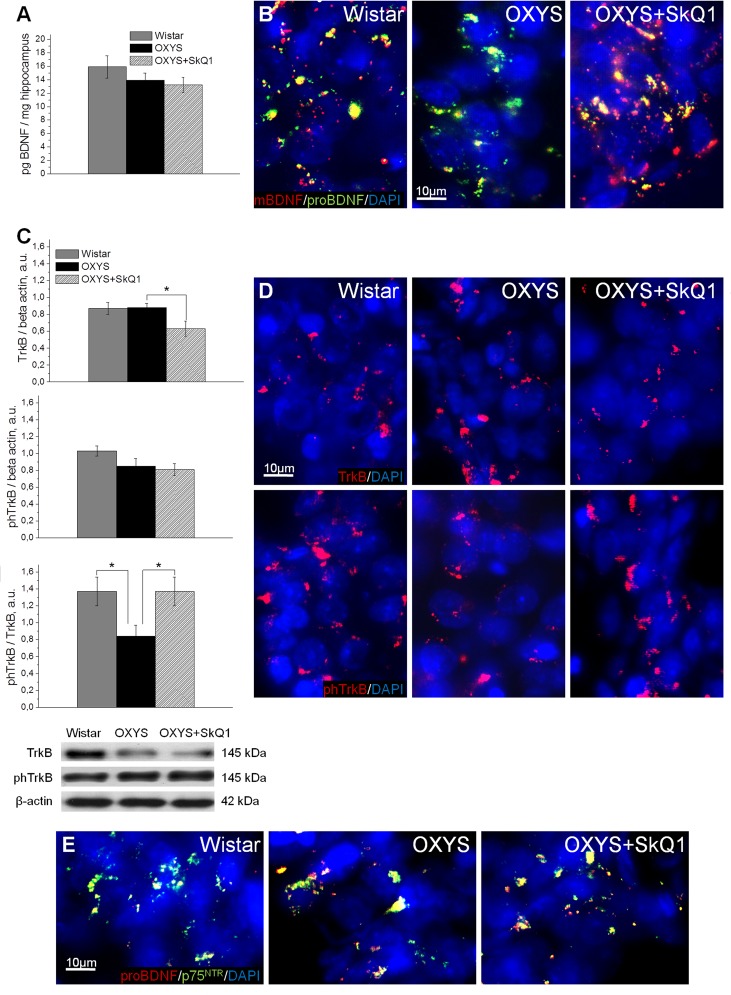
SkQ1 improves neurotrophic supply in the hippocampus (**A**) Enzyme immunoassay analysis showed no differences in the levels of total hippocampal BDNF among 18-month-old Wistar rats, untreated OXYS rats, and SkQ1-treated OXYS rats (n=8). (**B**) Representative 40 × immunofluorescent images of staining for mBDNF (red), proBDNF (green), and cell nuclei (DAPI, blue) in the CA1 region of the hippocampus. (**C**) Hippocampus levels of TrkB and phTrkB were not different between Wistar rats and untreated OXYS rats (n=6). The level of TrkB decreased in the hippocampus of SkQ1-treated OXYS rats (p<0.05). Measurement of the phTrkB/TrkB ratio showed a significant decrease of this ratio in the hippocampus of OXYS rats (p<0.04). Treatment with SkQ1 increased the phTrkB/TrkB ratio in OXYS rats (p<0.05). (**D**) Representative 40× immunofluorescent images of staining for receptors TrkB (red; upper row) and phTrkB (red; lower row) and for cell nuclei (DAPI, blue) in the CA1 region of the hippocampus. (**E**) Representative 40× immunofluorescent images of colocalization of proBDNF (red), p75^NTR^ receptor (green), and cell nuclei (DAPI, blue) in the CA1 region of hippocampus. A.u.: arbitrary units. The data are shown as mean ± SEM. Statistical significance (p<0.05) is denoted by the asterisk.

We next assessed the effects of SkQ1 treatment on the levels of brain-derived neurotrophic factor (BDNF) and of neurotrophic receptors tyrosine kinase B (TrkB) and p75 neurotrophin receptor (p75^NTR^), because we recently reported [32] that in OXYS rats, the cerebral level of BDNF compensatory increases in response to the development of neurodegenerative changes at early stages of AD-like pathology and dramatically decreases with age. Enzyme immunoassay analysis of BDNF (Fig. 4A) revealed no significant differences in protein levels of total BDNF between OXYS rats and Wistar rats. In addition, we found no effects of SkQ1 treatment on the BDNF protein level in OXYS rats. After that, we immunolabeled tissue samples with antibodies against the mature form of BDNF (mBDNF) and immature BDNF (proBDNF) to assess the contribution of each protein form to the total BDNF protein level in untreated or chronically SkQ1-treated OXYS rats and in Wistar rats (Fig. 4B). In the hippocampus, we found decreased mBDNF and increased proBDNF concentrations in OXYS rats and no differences in these proteins' levels between SkQ1-treated OXYS rats and Wistar rats. Therefore, the downregulation of mBDNF and upregulation of proBDNF in OXYS rats is indicative of a shift in the proBDNF/mBDNF balance in the direction of the immature form of the protein (proBDNF), which activates apoptosis.

Western blot analysis of the phospho-TrkB/TrkB ratio, which reflects activity of TrkB (a mBDNF receptor), revealed that in the OXYS hippocampus, this ratio was lower than that in Wistar rats (Fig. 4C). Immunolabeling of tissue samples with anti-TrkB and anti-phospho-TrkB antibodies confirmed this result (Fig. 4D). Moreover, immunolabeling for proBDNF and p75^NTR^ revealed increased colocalization of proBDNF with p75^NTR^, apparently caused by upregulation of proBDNF in the OXYS hippocampus (Fig. 4E). This result is suggestive of initiation of neuronal apoptosis, and as a consequence, of progression of neurodegenerative changes. SkQ1 treatment of OXYS rats increased the protein level of mBDNF and decreased that of proBDNF (Fig. 4B). Thus, the increase in the phospho-TrkB/TrkB ratio (Fig. 4C, D), downregulation of proBDNF, upregulation of mBDNF (Fig. 4B), and reduced immunoreactivity of p75^NTR^ as well as its colocalization with proBDNF (Fig. 4E) in the hippocampus of SkQ1-treated OXYS rats indicate activation of cellular processes promoting the growth of neurites, formation of new synapses, and neuronal survival.

### SkQ1 reverses synaptic deficits

**Figure 5:**
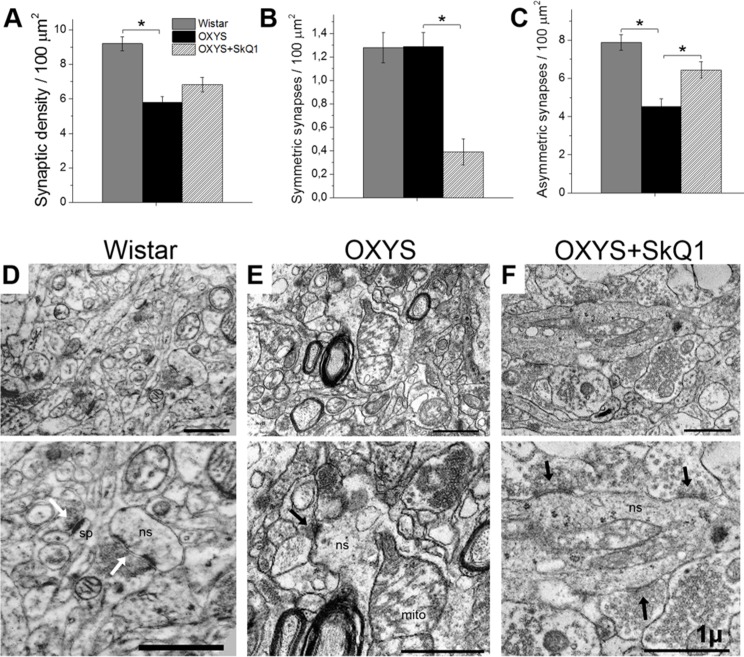
SkQ1 increased the density of asymmetric synapses and improved synaptic structure in the CA1 region of the hippocampus (**A**) The low density of synapses in the hippocampus of OXYS rats (n=4; p<0.05) and an increase by 17% in response to SkQ1 treatment. (**B**) A decrease in the number of symmetric synapses in response to SkQ1 treatment (p< 0.05). (**C**) The low number of asymmetric synapses in the hippocampus of OXYS rats and an increase in response to SkQ1 treatment (p<0.05). (**D–F**) Typical synaptic neuropil of the CA1 region in a Wistar rat, untreated OXYS rat, and SkQ1-treated OXYS rat. (**D**) Asymmetric synapses (white arrows) on a dendritic spine (sp) and on a nonspiny dendrite (ns) in a Wistar rat. (**E**) Symmetric synapse (black arrow) on a nonspiny dendrite (ns) containing a large mitochondrion (mito), and a degenerative myelinated axon in neuropil of an untreated OXYS rat. (**F**) Asymmetric synapses (black arrows) on a nonspiny dendrite (ns) in neuropil of an SkQ1-treated OXYS rat. The scale bar is 1 μm. The data are shown as mean ± SEM. Statistical significance (p<0.05) is denoted by an asterisk.

In line with their hippocampus-dependent cognitive deficits, OXYS rats show a synaptic loss [31], prominent alterations of synaptic functions [33], and significant ultrastructural changes [28] in the hippocampus. Ultrastructural abnormalities in synaptic terminals of OXYS rats in the period of progression of AD-like pathology are characterized by decreased numbers of synaptic vesicles, by signs of their disorganization and destruction, by increased numbers of various vacuoles, and by swelling and disintegration of mitochondria [31]. To determine whether chronic SkQ1 treatment alleviates synaptic deficits in OXYS rats, we measured SkQ1's effects on synaptic density in the CA1 region of the hippocampus. Electron-microscopic analysis showed that the synaptic density was significantly lower in OXYS rats (Fig. 5A) than in Wistar rats. Furthermore, the number of asymmetric (excitatory) synapses (classified by morphometric criteria) was almost twofold lower in OXYS rats (Fig. 5C) than in Wistar rats. In SkQ1-treated OXYS rats, the synaptic density was 17% higher (Fig. 5A) than that in untreated OXYS rats (insignificantly). In contrast, the number of excitatory synapses in SkQ1-treated OXYS rats was 42% greater (Fig. 5C) than that in untreated OXYS rats. The proportion of symmetric (inhibitory) synapses classified by morphometric criteria was twofold smaller in the SkQ1-treated OXYS rats than in untreated OXYS rats (Fig. 5B). In contrast to Wistar rats (Fig. 5D) and SkQ1-treated OXYS rats (Fig. 5F), in untreated OXYS rats, we observed degenerating synaptic terminals substantially more frequently (Fig. 5E); they showed characteristic clarification and swelling of the cytoplasm, disintegration of synaptic vesicles, an increase in the number of various vacuoles, and swelling and disintegration of mitochondria.

In addition, in SkQ1-treated OXYS rats, the share of excitatory synapses was 94% of total synaptic density, in untreated OXYS rats: 78%, and in Wistar rats: 86%.

**Figure 6:**
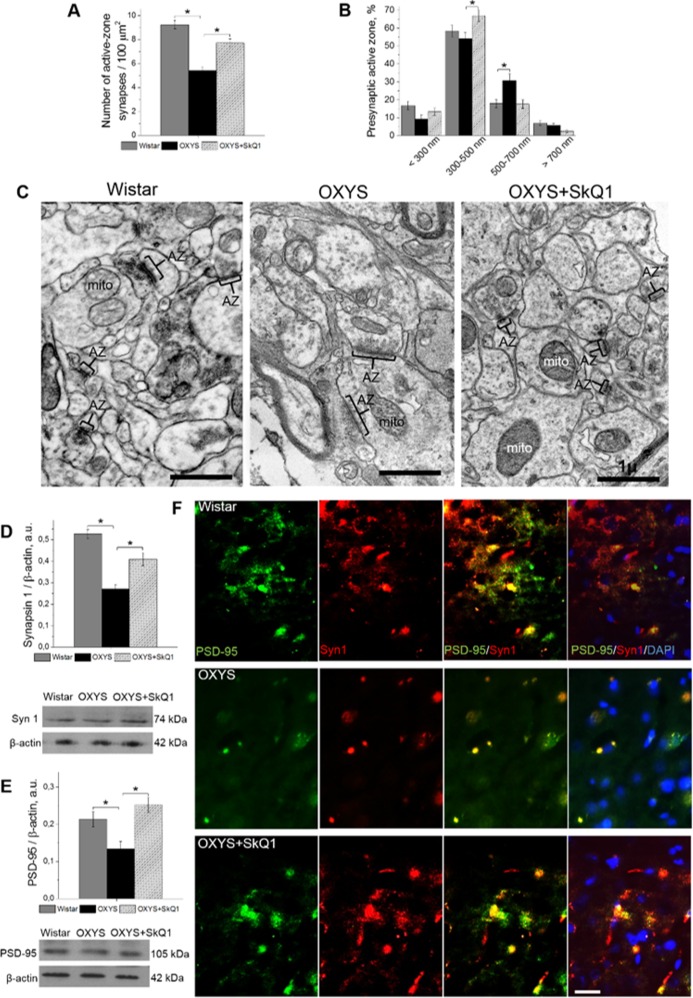
SkQ1 increased the number of presynaptic active zones in the hippocampal CA1 region and increased the levels of pre- and postsynaptic proteins in the hippocampus (**A**) The low number of presynaptic active zones in the hippocampus of OXYS rats (n=4; p<0.05) and an increase in response to SkQ1 treatment (p<0.05). (**B**) The elevated number of large presynaptic active zones (judging by the length of an active zone in the micrographs) in the hippocampus of OXYS rats (p<0.05) and an increase in the number of medium presynaptic active zones in response to SkQ1 treatment (p<0.05). (**C**) The electron micrographs show an active zone (AZ) in the CA1 region of the hippocampus in a Wistar rat and untreated and SkQ1-treated OXYS rats. (**D**, **E**) Synapsin I and PSD-95 levels were low in the hippocampus of untreated OXYS rats (p<0.05) and increased in response to SkQ1 treatment (p<0.05) according to western blot analyses (n=6-8). (**F**) Immunohistochemical staining (n=4) of synapsin I (red) and PSD-95 (green). The DAPI (blue) staining shows cell nuclei. The scale bar is 1 μm in (**C**) and 5 μm in (**F**). Mito: mitochondria, Syn I: synapsin I, a.u.: arbitrary units. The data are shown as mean ± SEM. Statistical significance (p<0.05) is denoted by an asterisk.

Accordingly, the proportion of active zones – a specialized portion of the presynaptic membrane to which synaptic vesicles dock and where they get primed for the release [34] – was greater in SkQ1-treated OXYS rats than in untreated OXYS rats (Fig. 6A). In this context, the increase in the proportion of large active zones in untreated OXYS rats (Fig. 6B) may reflect the processes of plastic reorganization directed at compensation for the regressive alterations of synaptic connections. Although in SkQ1-treated OXYS rats, the number of presynaptic active zones significantly increased (Fig. 6A, B), mostly due to medium active zones (300–500 nm), this change probably represents the mechanism of plastic reorganization of synapses and formation of new contacts among neurons. The result of the rearrangement of synaptic organization of the hippocampus in SkQ1-treated OXYS rats was an increased number of synaptic vesicles docked with the presynaptic membrane (Fig. 6C).

The conclusions of the morphometric assessment of the synaptic status were confirmed by the results of evaluation of SkQ1's influence on the hippocampal amounts of protein markers of pre- and postsynaptic density: synapsin I, participating in the regulation of the process of neurotransmitter release in synapses, and postsynaptic density protein 95 (PSD-95): the main protein of postsynaptic density. According to western blot analysis, the hippocampal level of both proteins in untreated OXYS rats was significantly lower (Fig. 6D, E) than that in Wistar rats. Prolonged administration of SkQ1 significantly increased the levels of synapsin I and PSD-95 in the hippocampus of OXYS rats (Fig. 6D, E), to the level of the Wistar strain. Finally, we immunolabeled tissue samples with anti-synapsin I and anti-PSD-95 antibodies to assess the effects of SkQ1 treatment on expression of the proteins (Fig. 6F). In the hippocampus, we observed decreased levels of synapsin I and PSD-95 in OXYS rats and no differences in these proteins' levels between SkQ1-treated OXYS rats and Wistar rats. Thus, these results indicate that chronic SkQ1 treatment probably not only ensured integrity of the existing neuronal connections but also promoted formation of new functionally active neuronal relations.

### SkQ1 decreases amyloid-β levels, tau hyperphosphorylation, and attenuates memory deficits

**Figure 7:**
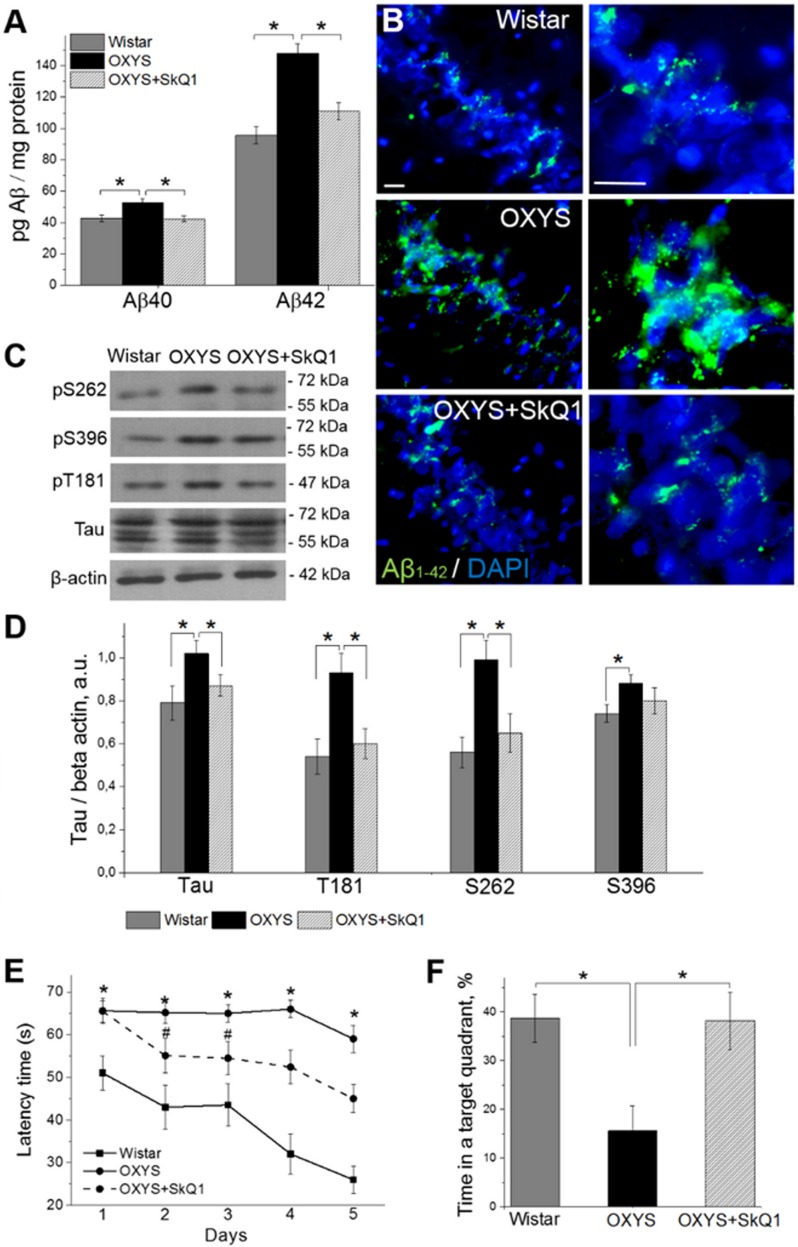
SkQ1 decreases amyloid-β levels and tau hyperphosphorylation and attenuates memory deficits (**A**) The increased levels of amyloid-β_1-40_ and amyloid-β_1-42_ in the hippocampus of untreated OXYS rats (n=8; p<0.05) were attenuated in response to SkQ1 treatment (p<0.05). (**B**) Immunostaining for amyloid-β_1-42_ (Aβ_1-42_ in green) in the hippocampus of Wistar rats, untreated OXYS rats, and SkQ1-treated OXYS rats. The DAPI (blue) staining shows cell nuclei. (**C** and **D**) The increased levels of Tau, pT181, pS262, and pS396 in the hippocampus of untreated OXYS rats (n=6; p<0.05) were significantly attenuated (except for pS396) in response to SkQ1 treatment (p<0.05). (**E**) Compared to Wistar rats, OXYS rats (n=8) showed increased escape latencies on all trial days (p<0.01) and (**F**) spent less time in the target quadrant on the sixth day (p<0.05). SkQ1 decreased escape latency of OXYS rats on the second trial day (p<0.02) and increased the time spent by OXYS rats in the target northwest quadrant on the sixth day (p<0.02). The scale bar is 5 μm. Aβ: amyloid-β, a.u.: arbitrary units. The data are shown as mean ± SEM. *p<0.05; ^#^p<0.05 for effects of SkQ1.

Because progressive accumulation of toxic amyloid-β species in the brain of OXYS rats starts at age 12 months [27–28], we tested whether treatment with SkQ1 since 12 months of age affects amyloid-β_1-40_ and amyloid-β_1-42_ levels in the hippocampus of 18-month-old OXYS rats. Enzyme immunoassay analysis of amyloid-β_1-40_ and amyloid-β_1-42_ (Fig. 7A) revealed significantly increased levels of these polypeptides in OXYS rats compared to Wistar rats. SkQ1 significantly lowered the protein levels of amyloid-β_1-40_ and amyloid-β_1-42_ in OXYS rats, to the level of Wistar rats. Finally, we immunolabeled tissue samples with an anti-amyloid-β_1-42_ antibody, to assess the influence of SkQ1 treatment on protein expression (Fig. 7B). In the hippocampus, we found increased amyloid-β_1-42_ levels in OXYS rats and no differences in amyloid-β_1-42_ content between SkQ1-treated OXYS rats and Wistar rats.

Because the levels of tau protein and of its phosphorylated form are increased in the brain of OXYS rats already at age 3 months [28], we then evaluated the ability of SkQ1 to slow down the hyperphosphorylation of tau protein in the hippocampus during progression of AD-like pathology. Western blot analysis of Tau and of its phosphorylated forms pT181, pS262, and pS396 (Fig. 7C, D) revealed significantly increased protein levels of all of the above in 18-month-old OXYS rats compared to Wistar rats. SkQ1 significantly lowered the protein expression of Tau, pT181, and pS262 in OXYS rats, to the level of Wistar rats (Fig. 7C, D).

Finally, we assessed hippocampus-dependent learning and memory of untreated or chronically SkQ1-treated OXYS rats in the Morris water maze as compared with Wistar rats. In the spatial (hidden-platform) component of the test, Wistar rats and SkQ1-treated OXYS rats learned better and faster than OXYS rats did (Fig. 7E). Moreover, untreated OXYS rats did not show any decrease in escape latency during the training. In addition, compared to Wistar rats, OXYS rats spent 2.5-fold less time in the target quadrant (Fig. 7F) on the sixth day (when the platform was removed); this is an indication of worsened reference memory. SkQ1-treated OXYS rats showed a significant decrease in escape latency already on the second trial day (Fig. 7E) and spent almost 2.5-fold as much time in the target quadrant on the sixth day (Fig. 7F) as compared to the untreated OXYS rats. Taken together, these data confirm our previous findings of increased amyloid-β levels, tau hyperphosphorylation, and cognitive deficits in OXYS rats at the progressive stage of AD-like pathology [28] and indicate that chronic SkQ1 treatment attenuates these processes, by decreasing amyloid-β levels and tau hyperphosphorylation and by restoring reference memory.

## DISCUSSION

The main purpose of this study was to assess the impact of mitochondrial dysfunction on the development of the sporadic form of AD using a nontransgenic rat model of this disease (the OXYS strain) and to test whether correction of mitochondrial dysfunction by means of the mitochondria-targeted antioxidant SkQ1 is an effective way to slow AD. According to our past and present data, we believe that the development and progression of AD-like pathology in OXYS rats may be caused by mitochondrial dysfunction. Five lines of evidence support this conclusion.

First, the development of early disturbances in mitochondrial function in OXYS rats is indicated by the significant increase in the prevalence of a common deletion (4834 bp) in mitochondrial DNA (mtDNA), especially at the stage of completion of brain development in the postnatal period [35]; this event is much earlier than accumulation of toxic forms of amyloid-β in the brain [28]. Meanwhile, the prevalence of mtDNA with the deletion in the hippocampus of OXYS rats stays elevated both at the stages preceding the development of signs of AD and during their progression. Accumulation of mtDNA deletions is considered one of the causes of the age-related decrease in the efficiency of oxidative phosphorylation. It also affects age-associated development of neurodegenerative diseases, including AD [36]. In particular, the lowered efficiency of the respiratory chain leads to enhanced accumulation of mtDNA deletions and oxidative damage to mtDNA; these processes may promote the buildup of toxic forms of amyloid-β, including because of the disruption in the production/clearance balance of this protein [37].

Second, the expression profile of mitochondrial genes in the brain of OXYS rats is significantly different from that of normal control rats [38], including the possible mitochondrial energy deficiency.

Third, enzymatic activity of complexes of the respiratory chain is decreased, and biosynthetic processes are aberrant in the hippocampus of old OXYS rats. Downregulation of oxidative phosphorylation and functional insufficiency of the fusion/fission system of mitochondria (associated with the development of AD) trigger mitochondrial disorders and activation of apoptotic processes. Both high activity of mitophagy in neurons and weakened elimination of mitochondria with structural or functional disturbances tend to shift the balance between functionally normal and defective mitochondria in favor of the latter [39–42].

Fourth, OXYS rats have (increasing with age) structural and functional mitochondrial alterations, detectable in various tissues and cells [20–22, 28, 31, 43–44], pointing to systemic disturbances in mitochondrial function; these problems can result in accelerated aging.

And fifth, treatment with the mitochondria-targeted antioxidant SkQ1 slows the development and progression of age-dependent diseases in OXYS rats [16–23], including AD-like pathology [24–25]. Recently, we showed that treatment of OXYS rats with SkQ1—starting at a young age—protected the rats from accumulation of mtDNA deletions in the hippocampus [35] and from neurodegeneration [25].

The main finding in the present study is that restoration of mitochondrial function by means of SkQ1 prevents and/or significantly slows the development of all signs of AD-like pathology in OXYS rats in the period of its active progression. We found that the proportion of damaged neurons in the hippocampus of SkQ1-treated OXYS rats was significantly smaller than that in untreated OXYS rats and was not different from this proportion in the Wistar strain. At the same time, according to analysis of the ultrastructural state of pyramidal neurons in the hippocampal CA1 region, in SkQ1-treated OXYS rats, there was a significant improvement of structural organization of organelles as well as increased specific area of mitochondria and endoplasmic reticulum, with decreased specific area of lysosomes and vacuoles. Our results revealed that SkQ1 did not affect the level of total BDNF in the OXYS hippocampus but upregulated mBDNF and downregulated proBDNF; these changes are suggestive of a shift in the proBDNF/mBDNF balance in favor of the mature form of the protein. Indeed, the increased phospho-TrkB/TrkB ratio and normalization of the balance of mature/immature forms of BDNF in the hippocampus of SkQ1-treated OXYS rats reflects activation of cellular processes promoting the growth of neurites, formation of new synapses, and neuronal survival.

The progression of AD-like pathology in OXYS rats is linked with substantial structural and functional alterations of synapses and with decreased synaptic density [31, 45]. Administration of SkQ1 not only ensured preservation of numerical density of synapses in the hippocampus of OXYS rats but, possibly, also facilitated activation of the remaining undamaged neurons and synapses and improved intraneuronal processes and axonal transport. This notion is supported by the increased proportion of asymmetric (excitatory) synapses and a greater number of active zones of synaptic contact as well as upregulation of pre- and postsynaptic proteins synapsin I and PSD-95, whose downregulation is considered an indicator event in AD [46–47].

We found that supplementation with SkQ1 in OXYS rats during active progression of AD-like pathology substantially improved their cognitive abilities, possibly as a result of the unique ability of SkQ1 to restore the processes of synaptic plasticity (as demonstrated in this study), not so much as a result of amyloid-β downregulation. Our hypothesis is that the pathogenesis of the familial form of AD differs from that of the sporadic form of this disease. We obtained additional evidence that the consequence of toxic accumulation of amyloid-β (in the brain of patients with the familial form of AD) is a disruption of AβPP processing, whereas in patients with the sporadic form of AD, the accumulation of amyloid-β can be mediated by synaptic processes [48]. Lately, the decisive role in progression of AD is attributed to trans-synaptic migration of toxic amyloid-β under the conditions of reorganization of interneuronal connections [49].

It should be emphasized that accumulation of amyloid-β in the brain of OXYS rats is a secondary event during the development of AD signs [28]. Manifestation of behavioral aberrations and deterioration of cognitive abilities in OXYS rats take place during mitochondrial dysfunction and hyperphosphorylation of tau protein [25, 27–28, 31]. Oxidative stress, chronic inflammation, and cellular stress are conducive to hyperphosphorylation of tau protein [50–53], whose consequence is disturbances of axonal transport [54]. These alterations can lead to destructive changes in axons, accumulation of damaged mitochondria there (mitochondrial abnormalities) and of other defective organelles [55–56], as we frequently saw in the hippocampus of 18-month-old untreated OXYS rats and in rare cases, in Wistar rats and in SkQ1-treated OXYS rats. Recently, in OXYS rats, we uncovered signs structural disturbances of myelin fibers of axons, structural changes in microtubules, and their disarray [31], which, to a substantial extent, can be a consequence of hyperphosphorylation of tau protein, as we demonstrated previously [25, 27–28] and in the present work. The neurotoxic action of tau protein can be mediated by amyloid-β, which promotes enhanced phosphorylation of tau protein and therefore, formation of neurofibrillary tangles via activation of kinases Cdk5 and GSK3b and by activating caspase 3, caspase 9, and calpain [57–58]. As shown in our previous studies, progression of the signs of AD in OXYS rats is accompanied by downregulation of the *Gsk3b* gene's mRNA in the cortex and increased mRNA expression of genes *Cdk5*, *Casp9*, and *Capn1*; these changes may also be triggered by the toxic forms of amyloid-β from neurodegenerative processes [38]. On the basis of the present results, we can hypothesize that the observed substantial improvement in the structure of myelin fibers and in the structure and organization of microtubules in SkQ1-treated OXYS rats are related to the ability of this antioxidant to attenuate the age-associated enhancement of tau protein phosphorylation; the latter improvement apparently led to normalization of transport pathways in cerebral neurons.

Studies involving primary neuronal cultures and hippocampal slices from AβPP transgenic mice showed that mitochondria-targeted antioxidants can prevent and/or slow down the amyloid-β-driven increase in ROS production, mitochondrial aberrations, disturbances of long-term post-tetanic potentiation, and neuronal death [59–61]. The ability of SkQ1 to slow down toxic effects of exogenous amyloid-β on formation of long-term post-tetanic potentiation in the hippocampus has also been demonstrated *in vitro* as well as *in vivo* [62]. Previously, we have shown that prophylactic dietary supplementation with SkQ1, starting before the manifestation of AD-like pathology (at the age of 1.5 months), significantly suppresses the accumulation of toxic forms of amyloid-β in the OXYS rats' brain and inhibits the development of all other signs of the disease [25]. Here, for the first time, we showed the ability of SkQ1 to suppress their development in elderly OXYS rats during active progression of AD-like pathology.

We cannot name the specific mechanisms of action of SkQ1 today, but the focus of this study is on the novel finding (by means of SkQR1) that dietary supplementation with SkQ1 allows this compound to penetrate and accumulate in the mitochondria of cerebral neurons. Earlier, effective penetration of SkQR1 into brain structures was shown only for its intranasal administration [63]. We can assume that the effects of SkQ1 are due to a direct antioxidant effect [64] as well as the ability to suppress mitochondrial production of ROS, and thereby to enhance the fatty-acid-driven mild decoupling of oxidation and phosphorylation [65]. We can also speculate that the effects of SkQ1, like those of another powerful neuroprotector, melatonin [31; 66], which are considered in recent years as a mitochondria-targeted antioxidant, are mediated by improved coordination of cellular signaling pathways responsible for tissue homeostasis [67]. In any case, this hypothesis is supported by the improved structural and functional state of mitochondria (and neurons on the whole) in SkQ1-treated OXYS rats as well as downregulation of amyloid-β and of tau protein hyperphosphorylation, recovery of synaptic processes and as a consequence, restoration of cognitive functions.

Thus, although the specific mechanisms of action of SkQ1 remain unclear, the fact that it specifically restores mitochondrial function and inhibits the development and progression of AD in a rat model is a compelling argument for its testing in clinical strategies targeting mitochondria for prevention and possibly treatment of this disease.

## MATERIALS AND METHODS

### Compounds

SkQ1 and SkQR1 (a rhodamine derivative of the former: SkQ1 decylrhodamine 19) were synthesized and provided by the Institute of Mitoengineering of Moscow State University (Moscow, Russia).

### Animal treatments

All experimental procedures were in compliance with the European Communities Council Directive of 24 November 1986 (86/609/EEC). The protocol of the animal study was approved by the Commission on Bioethics of the Institute of Cytology and Genetics, Novosibirsk, Russia. Male senescence-accelerated OXYS rats and Wistar rats were obtained from the Center for Genetic Resources of Laboratory Animals at the Institute of Cytology and Genetics, the Siberian Branch of the Russian Academy of Sciences (RFMEFI61914X0005 and RFMEFI61914X0010). The OXYS strain was derived from the Wistar strain of rats at the Institute of Cytology and Genetics as described earlier [24] and was registered in the Rat Genome Database (http://rgd.mcw.edu/). At this point, we have the 109^th^ generation of OXYS rats, with spontaneously developing cataract and accelerated senescence syndrome inherited in a linked manner.

At the age of 4 weeks, the pups were weaned, housed in groups of five animals per cage (57 × 36 × 20 cm), and kept under standard laboratory conditions (22°C ± 2°C, 60% relative humidity, and 12-hour light/12-hour dark cycle; lights on at 9 a.m.). The animals were provided with standard rodent feed (PK-120-1; Laboratorsnab, Ltd., Moscow, Russia) and water *ad libitum*.

To assess the influence of oral SkQ1 administration (from age 12 to 18 months) on progression of AD-like pathology, 12-month-old male OXYS rats were randomly assigned to one of two groups (n=15). One group consumed a control diet with addition of dried bread slices, and the other the same diet supplemented

with SkQ1 (250 nmol/[kg body weight]) on the dried bread slices. Each rat in the treatment group received SkQ1 daily. As controls, we used a group of Wistar rats (n=15).

To assess the localization and accumulation of SkQ1 in the brain by means of SkQR1, 4-month-old OXYS rats were randomly assigned to one of four groups (n=4). Two groups consumed a control diet supplemented with dried bread slices for 7 days or 14 days. The other two groups consumed the same diet supplemented with SkQR1 (250 nmol/kg) for 7 days or 14 days, respectively.

After treatment with SkQR1 in the 4.2- to 4.5-month-old cohorts, and after behavioral testing in the 18-month-old cohort, the rats were euthanized via CO_2_ asphyxiation. In both series of experiments, brains were carefully excised, and hemispheres were separated along the midline. For histological and immunohistochemical assays, the hemispheres were immediately fixed in 4% paraformaldehyde in phosphate-buffered saline. Fixed hemispheres were sliced at 5 or 20 μm (n=4-5; 4–6 serial tissue sections per animal) using a Microm HM-505 N cryostat. For western blot analysis and an enzyme-linked immunosorbent assay (ELISA), the hippocampus of 18-month-old rats (n=6-8) was separated from the brain, placed in a microcentrifuge tube for protein isolation, and frozen in liquid nitrogen. For assays of activities of mitochondrial complexes I and IV, the mitochondrial fraction was isolated from the rat hippocampus. For electron microscopic examination, the hippocampus samples (n=4) were fixed with 2.5% glutaraldehyde in sodium cacodylate buffer.

### Western blotting

Immunoblotting was performed as previously described [28]. Antibodies and dilutions used in this study include: anti-TrkB (1:1000; # ab18987, Abcam), anti-phospho-TrkB (phTrkB Y817; 1:1000; # ab81288, Abcam), anti-synapsin I (1:1000; # ab64581, Abcam), anti-PSD-95 (1:1000; # ab12093, Abcam), anti-tau (1:1000; # ab75714, Abcam), anti-phospho-tau (phospho T181; 1:1000; # ab75679, Abcam), anti-phospho-tau (phospho S262; 1:1000; # ab131354, Abcam), anti-phospho-tau (phospho S396; 1:1000; # ab109390, Abcam), and anti-β-actin (1:1000; # ab1801, Abcam). Quantitative densitometric analyses were performed on digitized images of immunoblots in the ImageJ software (NIH, USA).

### ELISAs

ELISAs for BDNF (BDNF [Rat] ELISA Kit; #KA0330, Abnova), for amyloid-β1-40 (Human/Rat ELISA Kit; # 294-62501, Wako), and for amyloid-β1-42 (Human/Rat ELISA Kit; # 290-62601, Wako) were performed on the isolated protein samples. Quantitation was carried out by measuring optical density on a microtiter plate reader, and the concentration was calculated in picograms of BDNF per milligram of hippocampal tissue or in picograms of amyloid-β_1-40_ or amyloid-β_1-42_ protein per milligram of total protein of hippocampal tissue.

### Assays of activity of mitochondrial complexes I and IV

Mitochondrial complex I activity was determined with the Complex I Rodent Enzyme Activity Microplate Assay Kit (# ab109721, Abcam) by quantifying oxidation of NADH to NAD+ and the simultaneous reduction of a dye, which causes an absorbance increase at 450 nm within 30 min using a CLARIO Star spectrophotometer (BMGLabtech). The Mitochondrial Complex IV Rodent Enzyme Activity Microplate Assay Kit (# ab109911, Abcam) was used to determine the activity of cytochrome c oxidase by quantifying oxidation of reduced cytochrome c using the absorbance change at 550 nm within 2 hours by means of CLARIO Star. The linear rate was examined, and enzymatic activity was expressed as an optic density (OD) alteration rate (OD/min) per μg of the isolated protein samples added per well.

### Histological examination

This analysis was performed as described elsewhere [31]. For the estimates of neuron numbers and to evaluate morphological features of the neurons in the middle molecular layer of the dentate gyrus, and CA1 and CA3 pyramidal layer, a set of 4–6 serial sections from each animal was used. A 100× objective lens (Axioplan 2, Zeiss) was used to count >200 neurons per visual field. The dead and damaged neurons were identified by their morphological features. The data were presented as a percentage of normal (no change), damaged, and dead neurons among all neurons in each visual field of a hippocampus sample. We measured the average area of the body and nucleus of the neurons per mm^2^. Images of the same region of the hippocampus were analyzed using the ImageJ software.

### Immunofluorescent staining

This procedure was performed by a standard indirect method as described previously [28]. Primary antibodies and dilutions were as follows: anti-COX IV (1:250; # ab16056, Abcam), anti-DRP1 (1:250; # ab56788, Abcam), anti-Mfn2 (1:250; # ab50838, Abcam), anti-proBDNF (1:250; # ab72440, Abcam), anti-mBDNF (1:250; # GF35L, Millipore), anti-TrkB (1:250; # ab18987, Abcam), anti-phospho-TrkB (phospho TrkB Y817; 1:250; # ab81288, Abcam), anti-p75^NTR^ (1:250; # ab93934, Abcam), anti-synapsin I (1:250; # ab64581, Abcam), anti-PSD-95 (1:250; # ab12093, Abcam), and anti-amyloid beta peptide (MOAB-2; 1:250; # MABN254, Millipore). After incubation with the respective secondary antibodies DyLight-650 or Alexa Fluor 488 (## ab96886, ab150073, Abcam) diluted 1:250, the slices were coverslipped with the Fluoro-shield mounting medium containing 4*′*,6-diamidino-2-phenylindole (DAPI; # ab104139, Abcam) and examined under an Axioplan 2 microscope (Zeiss).

### Electron-microscopic examination

The hippocampal samples of rats were prepared as described elsewhere [31]. For quantitative analysis, electron-transparent regions were identified on the electron micrographs of synapses and pyramidal neurons of the CA1 region (30 photos per animal). Then, all organelles that are located in these regions were painted using software. The photos were processed in Adobe Photoshop; for each photo, the following parameter was determined: the total area of each type of organelle located in the electron-transparent areas of the neurons. Then, the organelle-occupied proportion of the neuron area was calculated. We determined the number of interneuronal contacts (per visual field of 50 μm^2^) and calculated the numerical density of synapses per 100 μm^2^. Asymmetrical and symmetrical synapses were also counted. The synapses were classified (by the length of the active contact zone) into small (<300 nm), medium (300–500 nm), large (500–700 nm), and very large (>700 nm).

### Cognitive testing

The Morris water maze test [68] was used to analyze spatial memory: the rats (n=7) had to find a submerged platform in a pool of water, using external visual cues. Behavioral testing was carried out from 17.5 to 18 months of age as described previously [25].

### Statistical analysis

The data were subjected to ANOVA (Statistica 8.0 software). The Newman–Keuls test was applied to significant main effects and interactions in order to assess the differences between some sets of means. The data were presented as mean ± SEM. The differences were considered statistically significant at p<0.05.

## SUPPLEMENTARY MATERIAL FIGURES AND TABLE



## Abbreviations

APP: amyloid precursor protein
SkQ1: plastoquinonyl-decyltriphenylphosphonium
