# Supplementary material for: An antioxidant specifically targeting mitochondria delays progression of Alzheimer's disease-like pathology
Source: Aging (Albany NY). 2016 Oct 6;8(11):2713–31. doi: 10.18632/aging.101054 (PMC5191865; doi:10.18632/aging.101054)
Supplement: Supplementary file 1 [file aging-08-2713-s001.pdf]

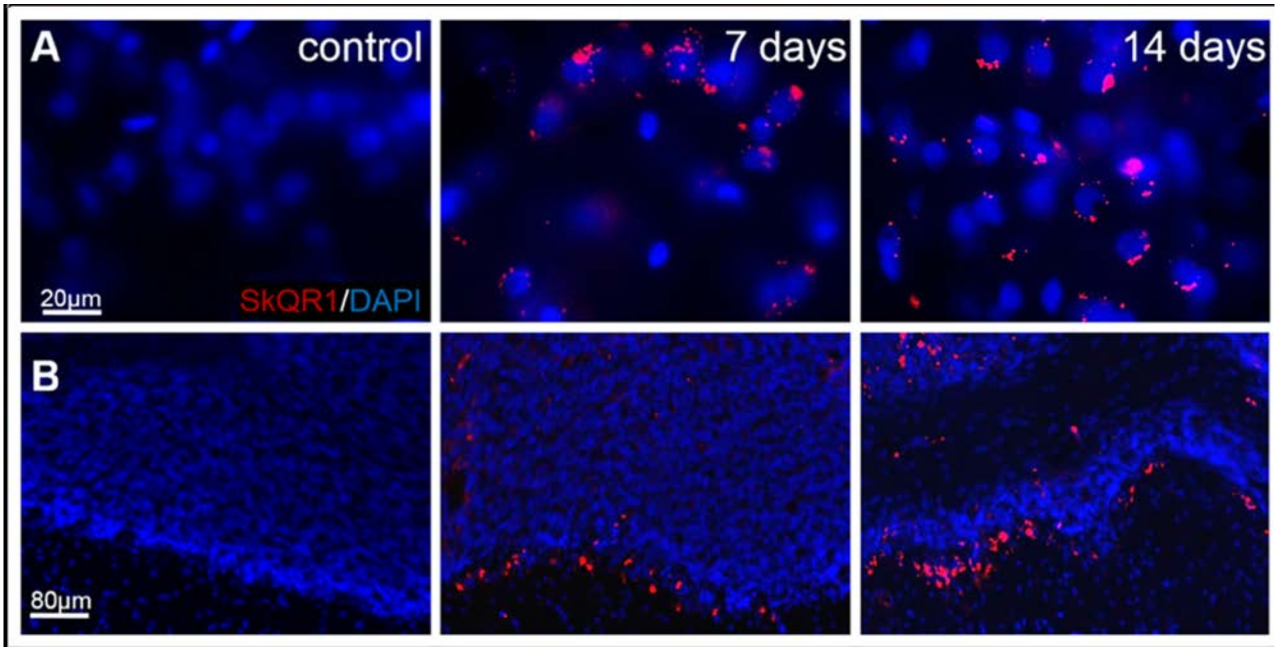

Figure S1. Accumulation of SkQ1 in the brain of OXYS rats. (**A** and **B**) Representative images showing accumulation of SkQR1 (a rhodamine derivative of SkQ1: SkQ1 decylrhodamine 19, red) in the frontal cortex (40×) and cerebellum (10×) of 4-month-old OXYS rats (n=4) depending on the duration of treatment. Cell nuclei are stained with DAPI (blue).

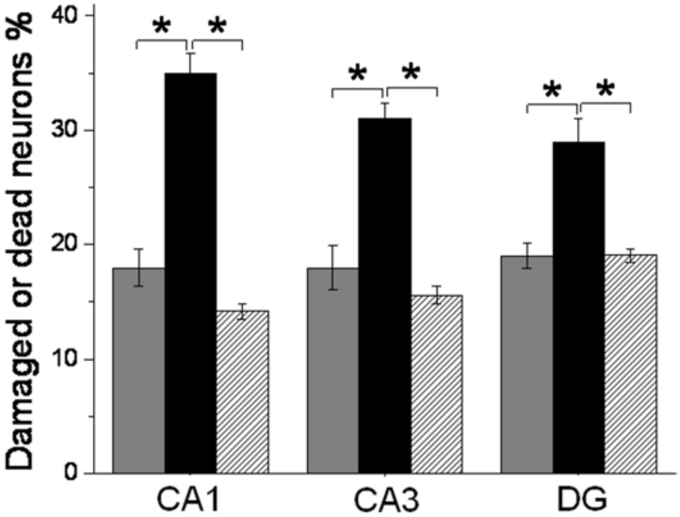

Figure S2. SkQ1 retards structural neurodegenerative alterations. The percentages of dead or damaged neurons in the examined hippocampal regions were greater in OXYS rats compared to Wistar rats. Oral SkQ1 administration improved neuronal health in all the hippocampal regions examined in OXYS rats. DG: dentate gyrus. The data are shown as mean ± SEM; *$p < 0.05$.

Table S1. The specific area of organelles in the cytoplasm of neurons (according to electron micrographs)

	Mitochondria, %	Lysosomes, %	Golgi apparatus, %	Rough ER, %	Vacuoles, %
Wistar	10.3±0.12	8.5±0.24	1.04±0.04	25.8±0.32	4.11±0.29
OXYS	3.1±0.1#	12.1±0.28#	1.19±0.03#	10.4±0.34#	8.58±0.32#
OXYS+SkQ1	7.8±0.16*	9.4±0.25*	1.08±0.05	18.1±0.42*	6.81±0.38

The data are presented as mean ± SEM (n=4). ER: endoplasmic reticulum. #$p < 0.05$ compared to Wistar rats; *$p<0.05$ compared to OXYS rats.
